# Circulating Sclerostin Levels Are Positively Related to Coronary Artery Disease Severity and Related Risk Factors

**DOI:** 10.1002/jbmr.4467

**Published:** 2021-12-09

**Authors:** Monika Frysz, Ingrid Gergei, Hubert Scharnagl, George Davey Smith, Jie Zheng, Deborah A Lawlor, Markus Herrmann, Winfried Maerz, Jon H Tobias

**Affiliations:** ^1^ Musculoskeletal Research Unit University of Bristol Bristol UK; ^2^ MRC Integrative Epidemiology Unit (IEU) University of Bristol Bristol UK; ^3^ Vth Department of Medicine (Nephrology, Hypertensiology, Rheumatology, Endocrinology, Diabetology) University Medical Center, Medical Faculty Mannheim, University of Heidelberg Mannheim Germany; ^4^ Therapeutic Area Cardiovascular Medicine Boehringer Ingelheim International GmbH Ingelheim Germany; ^5^ Clinical Institute of Medical and Chemical Laboratory Diagnostics Medical University of Graz Graz Austria; ^6^ Population Health Science, Bristol Medical School University of Bristol Bristol UK; ^7^ SYNLAB Academy SYNLAB Holding Deutschland GmbH Mannheim Germany

**Keywords:** LURIC, ALSPAC, CVD, SCLEROSTIN, DIABETES MELLITUS, HDL CHOLESTEROL

## Abstract

Romosozumab is a newly available treatment for osteoporosis acting by sclerostin inhibition. Its cardiovascular safety has been questioned after finding excess cardiovascular disease (CVD)‐related events in a pivotal phase 3 trial. Previous studies of relationships between circulating sclerostin levels and CVD and associated risk factors have yielded conflicting findings, likely reflecting small numbers and selected patient groups. We aimed to characterize relationships between sclerostin and CVD and related risk factors in more detail by examining these in two large cohorts, Ludwigshafen Risk and Cardiovascular Health study (LURIC; 34% female, mean age 63.0 years) and Avon Longitudinal Study of Parents and Children study (ALSPAC) mothers (mean age 48.1 years). Together these provided 5069 participants with complete data. Relationships between sclerostin and CVD risk factors were meta‐analyzed, adjusted for age, sex (LURIC), body mass index, smoking, social deprivation, and ethnicity (ALSPAC). Higher sclerostin levels were associated with higher risk of diabetes mellitus (DM) (odds ratio [OR] = 1.25; 95% confidence interval [CI] 1.12, 1.37), risk of elevated fasting glucose (OR 1.15; CI 1.04, 1.26), and triglyceride levels (β 0.03; CI 0.00, 0.06). Conversely, higher sclerostin was associated with lower estimated glomerular filtration rate (eGFR) (β −0.20; CI −0.38, −0.02), HDL cholesterol (β −0.05; CI −0.10, −0.01), and apolipoprotein A‐I (β −0.05; CI −0.08, −0.02) (difference in mean SD per SD increase in sclerostin, with 95% CI). In LURIC, higher sclerostin was associated with an increased risk of death from cardiac disease during follow‐up (hazard ratio [HR] = 1.13; 1.03, 1.23) and with severity of coronary artery disease on angiogram as reflected by Friesinger score (0.05; 0.01, 0.09). Associations with cardiac mortality and coronary artery severity were partially attenuated after adjustment for risk factors potentially related to sclerostin, namely LDL and HDL cholesterol, log triglycerides, DM, hypertension, eGFR, and apolipoprotein A‐I. Contrary to trial evidence suggesting sclerostin inhibition leads to an increased risk of CVD, sclerostin levels appear to be positively associated with coronary artery disease severity and mortality, partly explained by a relationship between higher sclerostin levels and major CVD risk factors. © 2021 The Authors. *Journal of Bone and Mineral Research* published by Wiley Periodicals LLC on behalf of American Society for Bone and Mineral Research (ASBMR).

## Introduction

Sclerostin is a WNT inhibitor secreted by osteocytes, which acts to inhibit osteoblast activity as part of bone's adaptive response to mechanical loading.^(^ ^1^ ^)^ Sclerostin was originally identified from studies of the high bone mass disorder sclerosteosis, caused by loss‐of‐function mutations in the *SOST* gene encoding sclerostin.^(^ ^2^ ^)^ Anti‐sclerostin antibody treatment has since been developed as a new treatment for osteoporosis acting by stimulating bone formation.^(^ ^3^ ^)^ Marketing authorization has recently been granted for the sclerostin antibody, romosozumab, to treat osteoporosis, after phase 3 trials indicating this is effective at reducing fracture risk.^(^ ^4^, ^5^ ^)^ However, use has been restricted because of concerns over cardiovascular safety, after an excess of cardiovascular events reported in the ARCH study^(^ ^5^ ^)^ as well as a phase 3 randomized trial in men with osteoporosis.^(^ ^6^ ^)^ Whether sclerostin inhibition impacts on cardiovascular disease (CVD) risk remains contentious. Whereas excess cardiovascular events were found in the romosozumab treatment arm compared with those treated with alendronic acid in the ARCH study^(^ ^5^ ^)^ and in men treated with romosozumab versus placebo,^(^ ^6^ ^)^ no excess was observed in the FRAME study, where the comparator group also consisted of placebo.^(^ ^4^ ^)^ These divergent findings may reflect a protective influence of bisphosphonates on CVD risk, as opposed to an adverse effect of romosozumab. Another bisphosphonate, zoledronate, has been found to decrease all‐cause mortality, to which reduced cardiovascular mortality may contribute.^(^ ^7^ ^)^ However, a beneficial effect on mortality was not born out in a meta‐analysis of drug trials of zoledronate and other bisphosphonates.^(^ ^8^ ^)^

Contrary to the suggestion that sclerostin inhibition may increase the risk of CVD, several studies have found lower sclerostin levels may protect against CVD. For example, higher sclerostin levels were found to be associated with higher risk of cardiovascular mortality in 98 chronic kidney disease (CKD) patients on peritoneal dialysis,^(^ ^9^ ^)^ 173 non‐dialysed CKD patients,^(^ ^10^ ^)^ and 130 participants with type 2 diabetes mellitus (T2DM)/prevalent CVD.^(^ ^11^ ^)^ On the other hand, higher sclerostin levels were found to be associated with reduced cardiovascular mortality in 673 renal dialysis patients.^(^ ^12^ ^)^ These findings are in line with previous studies indicating relationships between bone and cardiovascular disease.^(^ ^13^ ^)^ In terms of how sclerostin might influence CVD risk, sclerostin levels are related to a number of CVD risk factors. For example, sclerostin levels were reported to be higher in 40 T2DM patients compared with age‐matched controls,^(^ ^14^ ^)^ consistent with the recognized relationship between T2DM and fracture risk .^15^, ^16^, ^17^ Sclerostin levels have also been found to be increased in CKD patients, reflecting an inverse relationship between glomerular filtration rate (GFR) and sclerostin levels, which is not explained by reduced renal elimination.^(^ ^18^ ^)^

Though primarily produced by osteocytes, sclerostin has been detected in vascular tissue, including at sites of vascular calcification, suggesting sclerostin might also affect CVD risk as a consequence of a role in atherosclerotic plaque formation.^(^ ^19^, ^20^ ^)^ Consistent with this possibility, serum sclerostin levels have been found to be positively related to coronary artery calcification assessed by CT in 191 Afro‐Caribbean men^(^ ^21^ ^)^ and to predict arterial calcification from CT in 51 CKD patients undergoing dialysis.^(^ ^22^ ^)^ In contrast, sclerostin levels were found to be inversely related to carotid intima‐thickness (cIMT) in a combined group of 40 T2DM patients and 40 healthy controls^(^ ^23^ ^)^ and to (X‐ray‐based) aortic calcification in 207 hemodialysis patients.^(^ ^24^ ^)^

Therefore, observational studies suggest that sclerostin levels may be associated with a higher risk of vascular calcification and CVD and of associated risk factors such as T2DM and renal disease. However, much of the evidence is conflicting, likely reflecting small sample sizes and selected patient groups used in previous studies. To overcome these limitations, in the present study, we examined relationships between serum sclerostin and CVD and related risk factors, in the Ludwigshafen Risk and Cardiovascular Health (LURIC) and Avon Longitudinal Study of Parents and Children (ALSPAC) studies, together providing more than 5000 participants.

## Materials and Methods

### The Ludwigshafen Risk and Cardiovascular Health (LURIC)

The LURIC study is a prospective cohort study of individuals with and without cardiovascular disease and was designed to investigate environmental and genetic risk factors for the development of cardiovascular diseases. Between July 1997 and January 2000, 3316 participants of German ancestry were enrolled in the cardiology unit of a tertiary care medical center in southwestern Germany. The inclusion criteria comprised clinically stable conditions except for acute coronary syndromes (ACS), German ancestry, and availability of a coronary angiogram (indicated after standard clinical test diagnoses like chest pain and a positive, noninvasive stress test). Exclusion criteria were prespecified as any acute illness other than ACS, any chronic disease where noncardiac disease predominated, and a history of malignancy within the past 5 years. The detailed study protocol has been published.^(^ ^25^ ^)^ Of the originally recruited sample, 2054 participants (62%) had complete outcome and covariate data and provided the sample for our study. Written informed consent was obtained from each participant before inclusion. The study was in accordance with the Declaration of Helsinki and approved by the ethics committee at the Medical Association of Rhineland‐Palatinate (Ärztekammer Rheinland‐Pfalz).

CVD risk factors, namely T2DM, body mass index (BMI), hypertension, and smoking, were recorded at study entry. Socioeconomic status was defined using a proxy measure based on the regional purchasing index, collected at study entry as previously described.^(^ ^26^ ^)^ All patients were screened for T2DM at baseline, diagnosis being based on the 2014 criteria of the American Diabetes Association,^(^ ^27^ ^)^ history of T2DM, and/or use of oral anti‐diabetics or insulin. Cardiac‐related death was subsequently ascertained. Information on vital status was obtained from local registries. Two experienced physicians adjudicated independently the causes of death. In case of disagreement, one of the principal investigators of LURIC (WM) made the final assignment. Coronary artery stenosis (defined as >50% narrowing in one or more artery) and Friesinger score were evaluated on coronary angiograms as previously described.^(^ ^25^ ^)^ Fasting blood samples collected at baseline were used for measurement of glucose (converted to binary variable to indicate high/low measurement based on whole blood ≥6.1 mmol/L), triglycerides, low‐density lipoprotein (LDL) cholesterol, high‐density lipoprotein (HDL) cholesterol, apolipoprotein AI, apolipoprotein B, lipoprotein (a). The estimated glomerular filtration rate (eGFR) was calculated using the Chronic Kidney Disease Epidemiologic Collaboration (CKD‐EPI) formula. Hypertension was defined as a systolic blood pressure (BP systolic) ≥140 mmHg and a diastolic blood pressure (BP diastolic) ≥90 mmHg according to the European Society of Cardiology (ESC) guideline.^(^ ^28^ ^)^ Sclerostin was subsequently measured on baseline serum samples stored at minus 70°C using a chemiluminescent immunoassay (CLIA; DiaSorin LIAISON, DiaSorin, Stillwater, MN, USA).^(^ ^29^ ^)^ The assay was performed on a LIAISON XL Analyzer. The interassay coefficients of variation (CVs) were 4.3% and 5.2% at concentrations of 457 and 2460 pg/mL, respectively.

### Avon Longitudinal Study of Parents and Children

ALSPAC is a prospective birth cohort that recruited pregnant women with expected delivery dates between April 1991 and December 1992 from Bristol, UK. The initial number of pregnancies enrolled was 14,541. Of these initial pregnancies, there was a total of 14,676 fetuses, resulting in 14,062 live births and 13,988 children who were alive at 1 year of age. When the oldest children were approximately 7 years of age, an attempt was made to bolster the initial sample with eligible cases who had failed to join the study originally. The total sample size for analyses using any data collected after the age of 7 years is 15,454 pregnancies, resulting in 15,589 fetuses. Of these, 14,901 were alive at 1 year of age. Detailed information on the health and development of children and their parents were collected from regular clinic visits and completion of questionnaires.^(^ ^30^, ^31^ ^)^ Ethical approval was obtained from the ALSPAC Law and Ethics Committee and the Local Ethics Committees. The study website contains details of all the data that are available through a fully searchable data dictionary (http://www.bristol.ac.uk/alspac/researchers/our-data/).

The present study uses data from a research clinic undertaken between 2008 and 2011. All eligible mothers (ie, still engaged with the study; alive with known contact details and who had not withdrawn their consent) were invited to these assessments. Of 11,264 (77.5%) women invited, 4832 (43%) attended, of whom 3015 (62%) had complete outcome and covariate data and provided the sample for our study. cIMT (mm) of the left and right common carotid arteries were obtained via high‐resolution B ultrasound as previously described.^(^ ^32^ ^)^ Arterial distensibility was calculated as the difference between systolic and diastolic arterial diameters; the mean of left and right readings was used. Images were analyzed by a single trained reader.^(^ ^32^ ^)^

Height and weight were recorded at clinic attendance, and DM and hypertension ascertained from a questionnaire completed at the same time (whether responded as taking anti‐diabetic and/or high blood pressure medication) and complemented by questionnaire data completed between 2010 and 2012 (ever told they had DM/high blood pressure by a doctor). Smoking was ascertained from the same questionnaire collected between 2010 and 2012. For the purposes of this study, to optimize concordance with LURIC data, socioeconomic status was determined from postcodes available on file for enrolled mothers to derive Townsend score (1 = least deprived to 5 = most deprived). We used data linked between 2008 and 2011.

Fasting (minimum 6 hours) blood samples were collected at the time of clinic attendance and subsequently stored at–70°C. Fasting glucose was converted to binary variable to indicate high/low measurement (high glucose based on fasting plasma glucose concentration ≥7.0 mmol/L). Sclerostin was measured on heparin plasma blood samples using the Biomedica Human Sclerostin ELISA (BI‐20492, Biomedica Medizinprodukte, Vienna, Austria) kit. According to the manufacturer's protocol, the standard range for the array kit was 0 to 240 pmol/L and the lower detection limit was 3.2 pmol/L. Each kit was run with an internal quality control. All samples were run in duplicate and if the sclerostin measurement differed more than 20% between the duplicates, we removed the individual from further analysis. Outliers four standard deviations (SDs) away from the mean value were excluded. In our hands, intra‐assay CV% was 4.9%, and interassay CV% 11.3%. Standard clinical chemistry assays were performed for glucose, total and HDL cholesterol, and triglycerides, with LDL cholesterol estimated indirectly from these using Friedwalde's equation. A high‐throughput proton (1H) nuclear magnetic resonance platform was used to measure creatinine (from which eGFR was calculated^(^ ^33^ ^)^) and apolipoprotein A‐I and B measurements.

### Statistical methods

The distributions of continuously measured variables were explored using descriptive statistics and histograms. Lipoprotein(a) and triglycerides (TGs) were positively skewed and were log transformed for analysis so that residuals from the regression models were approximately normally distributed. Descriptive statistics are shown as means with standard deviations (SD) and medians with interquartile ranges (IQR) for continuous variables and counts with percentages (%) for categorical variables. All associations were analyzed using linear regression (for continuous outcomes), logistic regression (for binary outcomes), and Cox proportional hazards regression (for mortality data/death from cardiac cause). Association results are shown as differences in mean concentrations in SD units (continuous outcomes, including those that were log transformed), or odds ratios (OR)/hazard ratio (HR) (binary outcomes) per 1 SD higher sclerostin level, with 95% confidence intervals (CIs).

We divided adjustment variables into confounders (age, sex [LURIC], BMI, smoking, social deprivation, and ethnicity [ALSPAC] [model 2]) and potential mediators of sclerostin‐CVD relationships (LDL and HDL cholesterol, log triglycerides, diabetes, hypertension, eGFR, apolipoprotein A‐1). Associations between sclerostin and CVD risk factors were meta‐analyzed by combining summary statistics from model 2 in the two cohorts, employing random‐effects models due to the distinct characteristics of the two study populations, followed by tests for heterogeneity. All statistical analyses were performed in Stata 16.0.

## Results

Results were available from 2054 participants (34% female) from LURIC and 3015 from ALSPAC mothers (Supplemental Fig. S1; Tables 1 and 2). {TBL 1}{TBL 2} LURIC participants had a mean age of 63.0 years, of whom 76% had a clinical diagnosis of coronary artery disease (CAD), and 16% subsequently died as a result of cardiac disease over a mean follow‐up of 9.9 years. ALSPAC participants were considerably younger (mean 48.1 years). The majority of ALSPAC samples were of White ethnic group (95%), whereas all LURIC participants were White Europeans. LURIC participants had a higher prevalence of CVD risk factors compared with those from ALSPAC, with a greater proportion of those with DM (40.3% versus 1.8%), hypertension (72.8% versus 5.4%), and ever‐smokers (42.5% versus 40%), higher TG levels (167.5 versus 90.4 mg/dL), and lower HDL levels (39.2 versus 57.3 mg/dL). Differences in sclerostin levels between LURIC and ALSPAC participants are explained by the distinct assays used in the two cohorts.^(^ ^29^ ^)^

**Table 1:** Descriptives (Continuous Traits)

	LURIC						ALSPAC	
	Combined (*N* = 2054)		Males (*n* = 1350)		Females (*n* = 704)		(*N* = 3015)	
	Mean (SD)	IQR	Mean (SD)	IQR	Mean (SD)	IQR	Mean (SD)	IQR
Age (years)	63.0 (10.4)	(56:64:71)	62.0 (10.4)	(55:63:70)	64.9 (10.2)	(59:65:72)	48.1 (4.3)	(45:48:51)
Weight (kg)	79.4 (14.5)	(70:79:87)	83.8 (13.2)	(75:82:90)	71.1 (13.1)	(62:70:79)	71.1 (14.1)	(61:69:78)
Height (cm)	169.5 (8.7)	(164:170:176)	173.7 (6.7)	(169:173:178)	161.5 (6.4)	(157:162:166)	164.2 (6.1)	(160:164:168)
BMI (kg/m^2^)	27.6 (4.2)	(25:27:30)	27.7 (3.9)	(25:27:30)	27.2 (4.6)	(24:27:30)	26.4 (5.0)	(23:25:29)
Regional purchasing index	108.8 (7.7)	(104:108:112)	109.1 (7.8)	(104:108:113)	108.2 (7.6)	(102:107:111)		
Sclerostin (pg/mL)	385.9 (198)	(271:354:460)	411.7 (204)	(291:378:500)	336.3 (177)	(242:313:395)	1221 (500)	(871:1156:1502)
Triglycerides (mg/dL)	167.9 (111)	(106:143:197)	171.6 (119)	(109:144:196)	161 (93.3)	(101:139:200)	90.4 (48.1)	(60:79:105)
HDL cholesterol (mg/dL)	39.2 (10.7)	(32:38:45)	37.1 (9.5)	(30:36:43)	43.4 (11.6)	(35:42:50)	57.3 (15.0)	(46:56:66)
LDL cholesterol (mg/dL)	115.5 (34.4)	(93:113:137)	112.0 (32.4)	(90:111:133)	122.2 (37.1)	(98:120:144)	115.6 (31.0)	(94:113:134)
Apolipoprotein A‐I (mg/dL)	130.8 (25.6)	(112:129:147)	125.0 (23.0)	(109:124:140)	141.9 (26.7)	(123:140:159)	169.3 (17.3)	(157:168:180)
Apolipoprotein B (mg/dL)	104.1 (24.7)	(87:102:119)	103.2 (24.0)	(86:102:119)	105.7 (25.9)	(87:103:120)	86.1 (19.8)	(72:83:97)
Lipoprotein(a) (mg/dL)	30.1 (33.3)	(9:17:40)	29.3 (32.4)	(8:17:40)	31.5 (34.8)	(10:19:40)		
eGFR (mL/min)	85.3 (19.2)	(75:89:98)	86.7 (18.6)	(78:90:99)	82.7 (20.1)	(71:87:97)	99.3 (11.7)	(92:102:108)
Friesinger score	5.3 (3.9)	(2:5:8)	6.0 (3.9)	(3:6:9)	3.9 (3.5)	(1:3:7)		
cIMT (mm)							0.6 (0.1)	(0.52:0.56:0.57)
Average arterial distensibility (mm)							0.5 (0.1)	(0.42:0.49:5.42)

LURIC = Ludwigshafen Risk and Cardiovascular Health; ALSPAC = Avon Longitudinal Study of Parents and Children; SD = standard deviation; IQR = interquartile range (showing lower quartile, median, and upper quartile); BMI = body mass index; eGFR = estimated glomerular filtration rate; cIMT = carotid intima media thickness.

**Table 2:** Descriptives (Categorical Traits)

	LURIC (combined)	LURIC (males)		LURIC (females)	ALSPAC
Ethnic group	White	2054 (100)	1350 (100)	704 (100)	2948 (97.8)
	Non‐White	0	0	0	67 (2.2)
Townsend score	1				1184 (39.3)
	2				527 (17.5)
	3				558 (18.5)
	4				556 (18.4)
	5				190 (6.3)
Diabetes	No	1227 (59.7)	803 (59.5)	424 (60.2)	2962 (98.2)
	Yes	827 (40.3)	547 (40.5)	280 (39.8)	53 (1.8)
High glucose	No	1600 (77.9)	1053 (78.0)	547 (77.7)	2948 (97.8)
	Yes	454 (22.1)	297 (22.0)	157 (22.3)	67 (2.2)
Hypertension	No	558 (27.2)	394 (29.2)	164 (23.3)	2851 (94.6)
	Yes	1496 (72.8)	956 (70.8)	540 (76.7)	164 (5.4)
Smoking class	No	793 (38.6)	328 (24.3)	465 (66.1)	1820 (60.4)
	Ex	872 (42.5)	732 (54.2)	140 (19.9)	1195 (39.6)^a^
	Active	389 (18.9)	290 (21.5)	99 (14.1)	
Death from cardiac cause	No	1728 (84.1)	1122 (83.1)	606 (86.1)	
	Yes	326 (15.9)	228 (16.9)	98 (13.9)	
Death from any cause	No	1601 (78.0)	1035 (76.7)	566 (80.4)	
	Yes	453 (22.0)	315 (23.3)	138 (19.6)	
Coronary artery stenosis (*N* = 2023)	No	668 (33.0)	327 (24.5)	341 (49.4)	
	Yes	1355 (67.0)	1006 (75.5)	349 (50.6)	

LURIC = Ludwigshafen Risk and Cardiovascular Health; ALSPAC = Avon Longitudinal Study of Parents and Children.

Table shows frequency of characteristics (%).

^a^ Ex or active smoker.

**Fig. 1:**
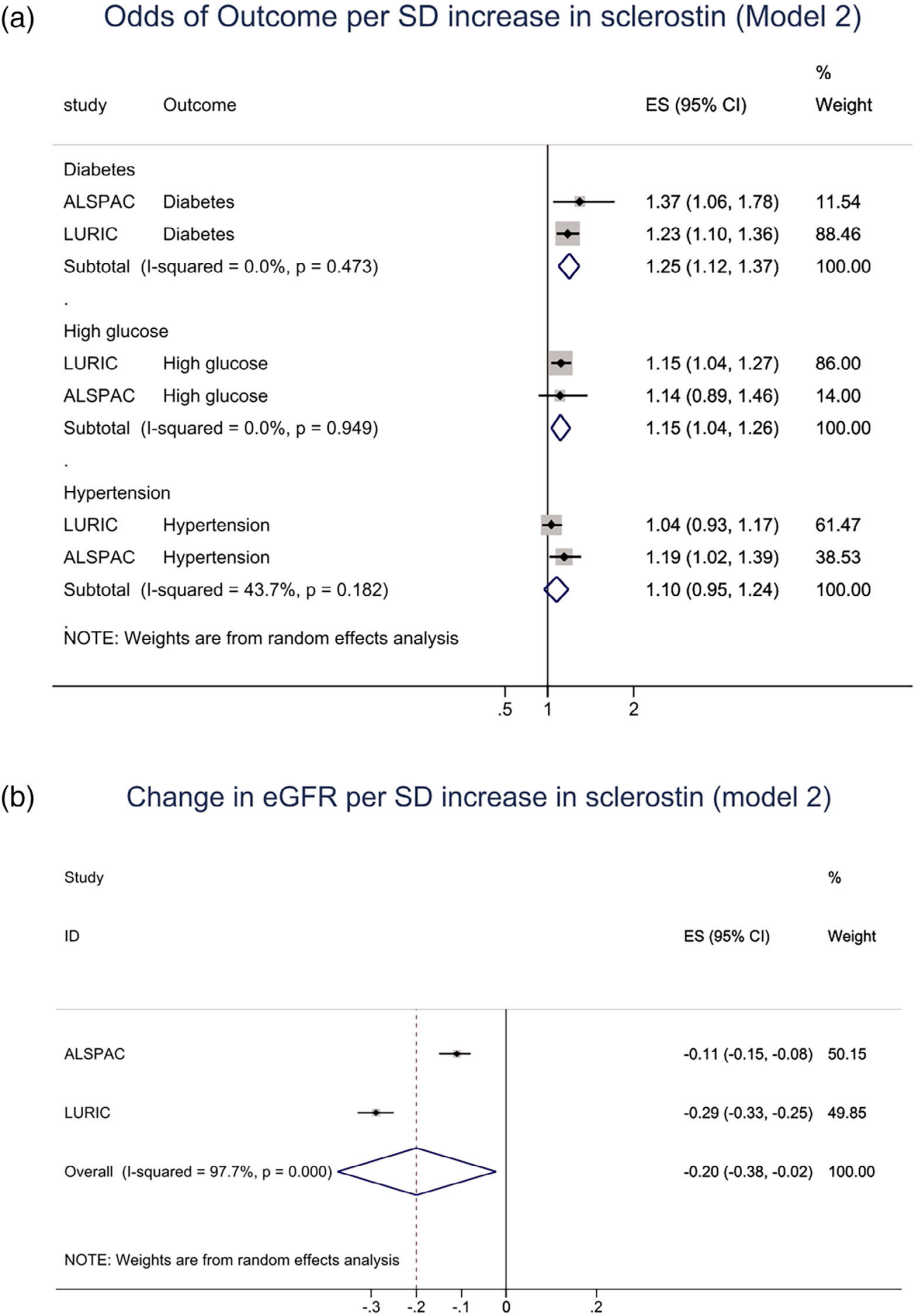
Meta‐analysis of associations between sclerostin and cardiovascular disease risk factors, adjusted for age and ethnic group (ALSPAC) and sex (LURIC), body mass index, smoking, and social deprivation. (*A*) Sclerostin and risk of diabetes (figure shows odds ratio per SD increase in sclerostin with 95% confidence intervals [CI]); high fasting glucose; risk of hypertension; (B) sclerostin and eGFR (SD change in eGFR per SD increase in sclerostin, with 95% CI).

We performed cross‐sectional analyses in LURIC and ALSPAC to examine associations between sclerostin levels and available CVD risk factors. Unadjusted and confounder‐adjusted (model 2) analyses revealed associations between sclerostin and eGFR, DM, and elevated fasting glucose, the strength of these associations varying in the individual cohorts (Table 3). {TBL 3} The relationship between sclerostin and eGFR appeared to be independent of DM, given this was unaffected by further adjustment for fasting glucose (Supplemental Table S2). In meta‐analyses combining confounder‐adjusted summary results from both cohorts, sclerostin was associated with a higher risk of DM (1.25; 1.12, 1.37) and elevated fasting glucose levels (1.15; 1.04, 1.26) (odds ratio per one SD change in exposure with 95% CI) and lower eGFR (−0.20; −0.38, −0.02) (change in standardized beta per one SD change in exposure with 95% CI) (Fig. 1*A*–*C*). {FIG1} Whereas unadjusted analyses showed a positive association between sclerostin and hypertension, this was largely attenuated after adjustment for confounders, and CIs overlapped the null in the meta‐analysis (Fig. 1*D*).

**Table 3:** Sclerostin Versus Clinical Risk Factors (ALSPAC/LURIC)

			LURIC (*N* = 2054)		ALSPAC (*N* = 3015)	
Exposure	Outcome	Model	β (95% CI)	*p*	β (95% CI)	*p*
Sclerostin	eGFR	1	−0.36 (−0.40, –0.31)	<0.001	−0.17 (−0.21, –0.14)	<0.001
Sclerostin	eGFR	2	−0.29 (−0.33, –0.25)	<0.001	−0.11 (−0.15, –0.08)	<0.001

			OR (95% CI)	*p*	OR (95% CI)	*p*
Sclerostin	Diabetes	1	1.39 (1.24, 1.55)	<0.001	1.31 (1.03, 1.68)	0.030
Sclerostin	Diabetes	2	1.23 (1.10, 1.36)	<0.001	1.37 (1.06, 1.78)	0.018
Sclerostin	High glucose	1	1.20 (1.08, 1.33)	<0.001	1.14 (0.90, 1.44)	0.272
Sclerostin	High glucose	2	1.15 (1.04, 1.27)	0.009	1.14 (0.89, 1.46)	0.286
Sclerostin	Hypertension	1	1.23 (1.09, 1.40)	0.001	1.24 (1.07, 1.43)	0.005
Sclerostin	Hypertension	2	1.04 (0.93, 1.17)	0.459	1.19 (1.02, 1.39)	0.030

LURIC = Ludwigshafen Risk and Cardiovascular Health; ALSPAC = Avon Longitudinal Study of Parents and Children; CI = confidence interval; eGFR = estimated glomerular filtration rate; OR = odds ratio.

Table shows results of linear/logistic regression analysis. Results are SD change in outcome/odds of outcome per SD increase in sclerostin, 95% CI, and *p* value. High glucose based on fasting plasma glucose concentration ≥7.0 mmol/L (whole blood ≥6.1 mmol/L). Model 1: unadjusted; model 2: adjusted for age and ethnic group (ALSPAC) and sex (LURIC), body mass index, smoking, and social deprivation.

**Fig. 2:**
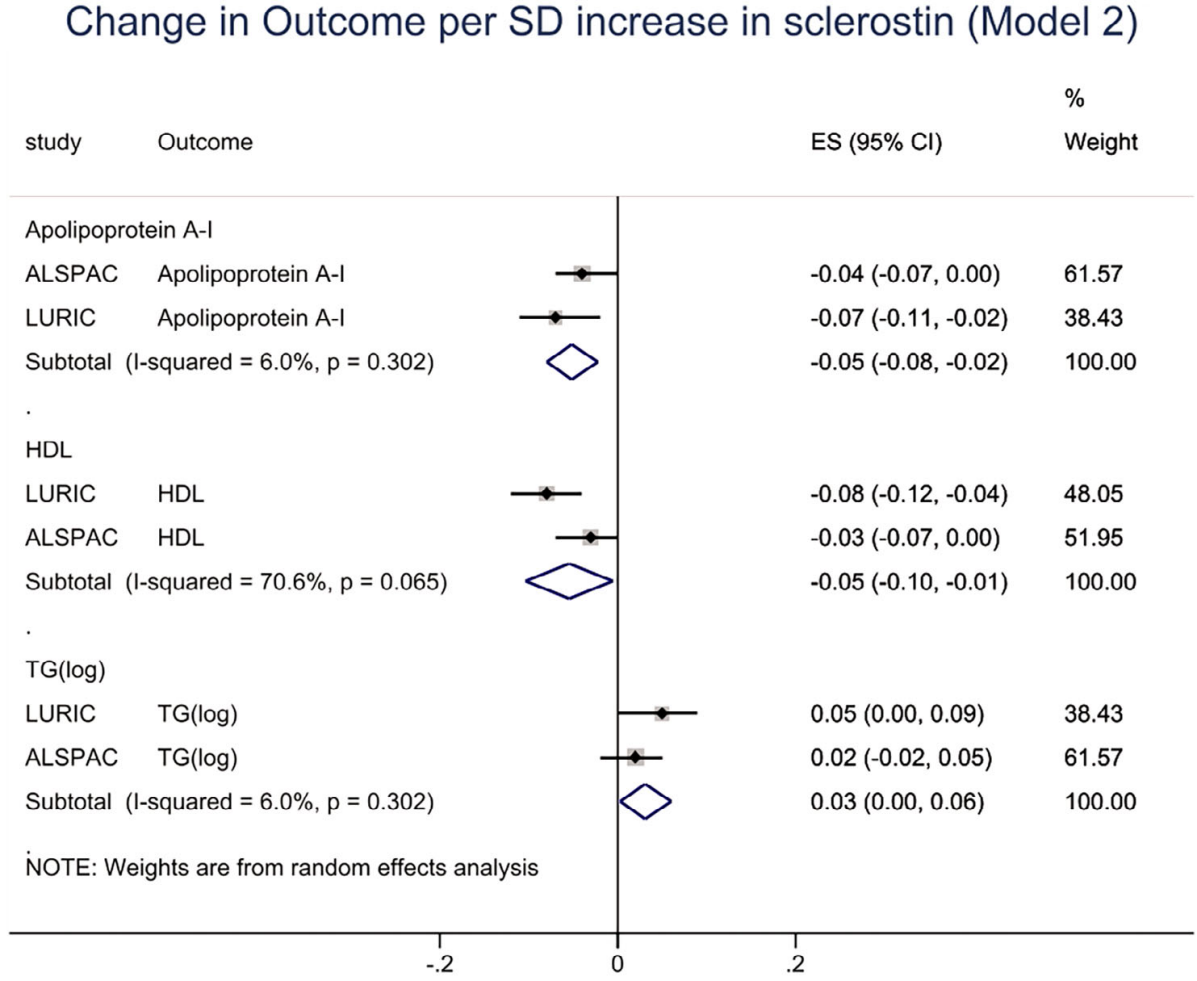
Meta‐analysis of associations between sclerostin and cardiovascular disease risk factors, adjusted for age and ethnic group (ALSPAC) and sex (LURIC), body mass index, smoking, and social deprivation. Figure shows sclerostin vs. apolipoprotein A‐I, HDL and triglyceride levels (TG)(log) (SD change in outcome per SD increase in sclerostin, with 95% confidence intervals).

We also analyzed associations between sclerostin and lipid fractions. In confounder‐adjusted analyses, sclerostin levels were positively related to TGs in LURIC, though little evidence of association was found in ALSPAC (Table 4). {TBL 4} However, meta‐analyses combining results from both cohorts revealed a weak positive association between sclerostin and TGs (0.03; 0.00, 0.06) (Fig. 2*A*). {FIG2} Sclerostin was inversely related to HDL cholesterol, in both LURIC and ALSPAC alone, and in meta‐analyses combining both cohorts (−0.05; −0.10, −0.01) (Fig. 2*B*). Sclerostin was also inversely related to apolipoprotein A‐1, in both cohorts and the meta‐analysis (Fig. 2*C*). No consistent associations were found with LDL cholesterol, apolipoprotein B, or lipoprotein(a) (Table 4). We also analyzed associations between categorical levels of sclerostin (split into quartiles) and outcomes, findings generally suggesting dose–response relationships (Supplemental Figs. S2–S5). In addition, results are included stratified by smoking and BMI (ALSPAC; Supplemental Figs. S6–S9) and sex (LURIC; Supplemental Figs. S10 and S11).

**Table 4:** Sclerostin Versus Lipids (ALSPAC/LURIC)

			LURIC (*N* = 2054)		ALSPAC (*N* = 3015)	
Exposure	Outcome	Model	β (95% CI)	*p*	β (95% CI)	*p*
Sclerostin	Triglycerides (log)	1	0.03 (−0.01, 0.07)	0.173	0.04 (0.01, 0.08)	0.024
Sclerostin	Triglycerides (log)	2	0.05 (0.00, 0.09)	0.038	0.02 (−0.02, 0.05)	0.330
Sclerostin	LDL	1	−0.05 (−0.09, –0.01)	0.021	0.05 (0.01, 0.09)	0.007
Sclerostin	LDL	2	−0.02 (−0.07, 0.02)	0.337	0.00 (−0.03, 0.04)	0.877
Sclerostin	HDL	1	−0.11 (−0.15, –0.07)	<0.001	−0.01 (−0.05, 0.02)	0.513
Sclerostin	HDL	2	−0.08 (−0.12, –0.04)	<0.001	−0.03 (−0.07, 0.00)	0.057
Sclerostin	Apolipoprotein A‐I	1	−0.11 (−0.15, −0.07)	<0.001	0.00 (−0.04, 0.03)	0.935
Sclerostin	Apolipoprotein A‐I	2	‐0.07 (−0.11, –0.02)	0.002	−0.04 (−0.07, 0.00)	0.026
Sclerostin	Apolipoprotein B	1	−0.01 (−0.05, 0.03)	0.665	0.06 (0.02, 0.09)	0.002
Sclerostin	Apolipoprotein B	2	0.01 (−0.03, 0.06)	0.525	0.01 (−0.02, 0.05)	0.464
Sclerostin	Lipoprotein(a) (log)	1	−0.03 (−0.07, 0.02)^a^	0.233		
Sclerostin	Lipoprotein(a) (log)	2	−0.02 (−0.06, 0.03)^a^	0.432		

LURIC = Ludwigshafen Risk and Cardiovascular Health; ALSPAC = Avon Longitudinal Study of Parents and Children; CI = confidence interval.

Table shows results of linear regression analysis. Results are SD change in outcome per SD increase in sclerostin, 95% CI, and *p* value. Model 1: unadjusted; model 2: adjusted for age and ethnic group (ALSPAC) and sex (LURIC), body mass index, smoking, and social deprivation.

^a^ Based on *n* = 1927.

Subsequently, we examined associations between sclerostin and CVD outcomes. In LURIC, unadjusted analyses showed strong associations between sclerostin and CVD‐related outcomes; however, these were partially attenuated after adjustment for confounders, reflecting associations between sclerostin and sex, age, and smoking (Supplemental Table S1). In confounder‐adjusted analyses, sclerostin was associated with an increased risk of death from cardiac disease during follow‐up (1.13; 1.03, 1.23) (Table 5). {TBL 5} In addition, sclerostin was positively associated with severity of coronary artery disease on angiogram at study entry, as reflected by Friesinger score (0.05; 0.01, 0.09); there was also evidence that sclerostin was associated with an increased risk of coronary artery stenosis (1.16; 1.02, 1.32). In ALSPAC, there were too few events to examine associations between sclerostin and clinical endpoints related to CVD. In confounder‐adjusted analyses, weak positive associations were observed between sclerostin and carotid intimal thickness and carotid artery distensibility, with wide 95% CIs that included the null.

**Table 5:** Sclerostin Versus CVD Disease Outcomes (LURIC/ALSPAC)

	LURIC (*N* = 2054)			
Exposure	Outcome	Model	β (95% CI)	*p*
Sclerostin	Friesinger score	1	0.14 (0.09, 0.18)	<0.001
Sclerostin	Friesinger score	2	0.05 (0.01, 0.09)	0.018
Sclerostin	Friesinger score	3	0.03 (−0.02, 0.07)	0.252

			HR (95% CI)	*p*
Sclerostin	Death from cardiac cause	1	1.21 (1.14, 1.28)	<0.001
Sclerostin	Death from cardiac cause	2	1.13 (1.03, 1.23)	0.007
Sclerostin	Death from cardiac cause	3	1.03 (0.93, 1.15)	0.557

			OR (95% CI)	*p*
Sclerostin	Coronary artery stenosis^a^	1	1.47 (1.29, 1.67)	<0.001
Sclerostin	Coronary artery stenosis^a^	2	1.16 (1.02, 1.32)	0.026
Sclerostin	Coronary artery stenosis^a^	3	1.09 (0.96, 1.24)	0.189

	ALSPAC (*N* = 3015)			
	Outcome		β (95% CI)	
Sclerostin	cIMT	1	0.06 (0.03, 0.10)	0.001
Sclerostin	cIMT	2	0.02 (−0.02, 0.05)	0.409
Sclerostin	cIMT	3	0.01 (−0.02, 0.05)	0.550
Sclerostin	Av. distensibility	1	−0.02 (−0.05, 0.02)	0.328
Sclerostin	Av. distensibility	2	0.02 (−0.01, 0.06)	0.183
Sclerostin	Av. distensibility	3	0.03 (−0.01, 0.07)	0.106

CVD = cardiovascular disease; LURIC = Ludwigshafen Risk and Cardiovascular Health; ALSPAC = Avon Longitudinal Study of Parents and Children; CI = confidence interval; HR = hazard ratio; OR = odds ratio; cIMT = carotid intima media thickness.

Table shows results of linear/logistic/Cox proportional hazards regression analysis. Results are SD change in outcome/odds/HR of outcome per SD increase in sclerostin, 95% CI, and *p* value. Model 1: unadjusted; model 2: adjusted for age and ethic group (ALSPAC) and sex (LURIC), body mass index, smoking, and social deprivation; model 3: model 2 plus LDL and HDL cholesterol, log triglycerides, diabetes, hypertension, estimated glomerular filtration rate, and apolipoprotein A‐I.

^a^ Based on *n* = 2023.

Finally, we explored whether associations between sclerostin and CVD risk factors contribute to the relationship between sclerostin and CAD observed in LURIC. Additional adjustment for available possible mediators (ie, DM, hypertension, eGFR, TGs, and LDL and HDL cholesterol; model 3) led to partial attenuation of the associations between sclerostin and Friesinger score, death from cardiac cause, and coronary artery stenosis, as reflected by 40%, 77%, and 44% decreases in effect estimates respectively (Table 5).

## Discussion

We examined associations between sclerostin levels and CVD outcomes and related risk factors in 5069 participants from the LURIC and ALSPAC cohorts. In confounder‐adjusted analyses in LURIC, sclerostin was positively related to CAD severity at study entry, as reflected by Friesinger score and coronary artery stenosis, and with cardiac mortality after follow‐up for a mean of 9.9 years. In meta‐analyses in both cohorts of summary data for possible mediators, sclerostin was positively related to the risk of DM and high fasting glucose level, and inversely related to eGFR, HDL cholesterol, and apolipoprotein A‐I. Associations between sclerostin and CVD in LURIC were partially attenuated by further adjustment for possible mediators, suggesting the latter may contribute to the relationship between sclerostin and CVD but do not explain these entirely.

We are not aware of any previous studies of relationships between sclerostin levels and either cardiac death or coronary angiogram findings. That said, our findings are broadly consistent with a positive relationship between sclerostin and CVD mortality observed in 130 participants with T2DM/prevalent CVD over a median follow‐up duration of 9.9 years.^(^ ^11^ ^)^ Previous studies have also examined associations between sclerostin levels and CVD mortality in CKD patients, with conflicting findings.^(^ ^9^, ^10^, ^12^ ^)^ The positive relationship observed between sclerostin and CAD severity in LURIC is consistent with previous observations that sclerostin is associated with an increased risk of coronary artery calcification on CT in Afro‐Caribbean men^(^ ^21^ ^)^ and in CKD patients undergoing dialysis.^(^ ^22^ ^)^ In contrast, in the former study, sclerostin was unrelated to aortic artery calcification, suggesting sclerostin may vary in its association with atherosclerosis at distinct vascular beds.^(^ ^21^ ^)^

The lack of association between sclerostin and cIMT in ALSPAC mothers likely reflects the fact that little variation in cIMT was observed, presumably because this comprises a relatively low‐risk group for CVD, as supported by the lower prevalence of CVD risk factors compared with LURIC. Differences in risk factor distributions between the two studies are also potentially influenced by differences in the populations, with LURIC participants selected on the basis of having high risk for CAD, and risk factor assessment. For example, DM classification in ALSPAC was based on a questionnaire asking about medication and previous diabetes diagnosis, whereas in LURIC, this was based on screening participants for elevated glycosylated hemoglobin and/or fasting blood sugar.

Our finding that sclerostin was inversely related to eGFR is consistent with previous reports that sclerostin levels are elevated in end‐stage renal disease, where they have been suggested to contribute to the development of chronic kidney disease mineral and bone disease (CKD‐MBD).^(^ ^34^ ^)^ Equivalent inverse relationships between sclerostin and eGFR were previously reported across the full range of CKD classes^(^ ^18^ ^)^ and in an unselected group of 352 Japanese postmenopausal women.^(^ ^35^ ^)^ Whereas renal retention of sclerostin as GFR declines may partly explain this relationship, increasing osteoblast production with reducing GFR has also been suggested to contribute.^(^ ^36^ ^)^

Our finding that DM was associated with higher sclerostin levels likely reflected an association with T2DM, which comprised DM cases in LURIC, and presumably explains the great majority of ALSPAC cases (although ascertainment of DM in ALSPAC was based on use of anti‐diabetic medication, which would have included those with T1DM, 90% of UK adults with DM have T2DM^(^ ^37^ ^)^). Therefore, our results are consistent with those from previous case‐control studies reporting higher sclerostin levels in T2DM patients.^(^ ^14^, ^38^ ^)^ Whereas Gennari and colleagues found that sclerostin was elevated in adults with T2DM, but not those with T1DM,^(^ ^14^ ^)^ higher sclerostin levels have been reported in adolescent girls with T1DM.^(^ ^39^ ^)^ As well as contributing to relationships with CVD, elevated sclerostin in T2DM may play a role in osteoporosis, leading to increased fracture risk.^(^ ^40^ ^)^ Although the mechanisms linking sclerostin to T2DM are currently unclear, the WNT pathway is also known to have a role in regulating adipogenesis,^(^ ^41^ ^)^ which is likely to impact on insulin sensitivity and hence risk of T2DM. Additionally, sclerostin is thought to stimulate bone resorption via a RANKL‐dependent pathway,^(^ ^42^ ^)^ which is predicted to release undercarboxylated osteocalcin from the bone matrix. Undercarboxylated osteocalcin has been shown to stimulate pancreatic insulin secretion, resulting in improved glucose homeostasis and DM risk.^(^ ^43^, ^44^, ^45^ ^)^

In terms of a possible role of altered lipid metabolism in mediating relationships between sclerostin and CVD, we found an inverse association between sclerotin and HDL cholesterol but not other forms of cholesterol. Consistent with this observation, there was also evidence that sclerostin was inversely related to apolipoprotein A1, the main lipoprotein associated with HDL cholesterol. Given the established inverse relationship between HDL and risk of CVD and CAD in particular,^(^ ^46^ ^)^ this finding could certainly contribute to the association we observed between sclerostin and CAD severity. A similar inverse relationship between sclerostin and HDL cholesterol was observed in Japanese postmenopausal women, although in contrast to the present findings, this study also reported a positive relationship between sclerostin and LDL cholesterol.^(^ ^35^ ^)^

The finding that adjustment for these risk factors partially attenuated the association between sclerostin and CAD severity suggests that they contributed to this relationship but did not explain it entirely. This raises the possibility that sclerostin might also exert direct actions on vascular calcification and atherosclerosis, consistent with previous observations that sclerostin is present in vascular tissue, including at sites of vascular calcification.^(^ ^19^, ^20^ ^)^ Alternatively, since higher sclerostin expression is associated with accumulation of glycation end‐products (AGEs) in bone tissue of diabetic patients,^(^ ^47^ ^)^ and given the ubiquitous nature of AGE accumulation in diabetes including in cardiovascular tissue,^(^ ^48^ ^)^ this might represent a further mechanism by which sclerostin influences risk of CVD. However, as discussed below, mediation analyses in observational studies need to be treated with caution. As for the biological mechanisms involved in possible direct effect of sclerostin on atherosclerosis, WNT signaling has previously been suggested to contribute to the development of atherosclerosis.^(^ ^49^ ^)^ However, as a WNT inhibitor, sclerostin would be predicted to protect against atherosclerosis, as opposed to increasing its risk. Although sclerostin acts to inhibit skeletal mineralization, this is secondary to its effect on bone formation and osteoblast function and represents an entirely distinct process to vascular calcification.

It is not possible to attribute causality given the largely cross‐sectional nature of our study, and it is equally plausible that our findings represent a causal effect of CAD severity on sclerostin levels, perhaps representing a physiological adaptation to vascular calcification.^(^ ^49^ ^)^ Another possibility is that certain medications associated with CAD severity and/or risk factors have been found to be associated with BMD and might also be related to sclerostin levels and hence contribute to a possible causal effect of CAD on sclerostin levels. These include anti‐diabetic drugs such as thiazolidinediones, which have previously been reported to accelerate bone loss and increase the risk of fractures.^(^ ^50^ ^)^ Unfortunately, we were unable to examine this question further because we did not have access to information about use of specific anti‐diabetic agents.

Mendelian randomization can be used to examine causality; however, although we used this approach to examine causal effects of circulating sclerostin on bone mineral density (BMD) and fracture risk, we had insufficient power to examine relationships with CVD risk.^(^ ^51^ ^)^ Two recent studies used a genetics approach to examine causal relationships between sclerostin inhibition and CVD, following the selection of *SOST* genetic variants without reference to circulating sclerostin levels. Bovijn and colleagues selected two *SOST* single‐nucleotide polymorphisms (SNPs) on the basis of associations with BMD and sclerostin expression in human osteoblast cultures, which were found to be associated with an increased risk of CVD and related risk factors such as T2DM and hypertension.^(^ ^52^ ^)^ Conversely, Holdsworth and colleagues selected variants in the *SOST* region based on associations with reduced *SOST* expression in arterial or heart tissue and increased BMD, which showed no association with CVD‐related outcomes in UK Biobank.^(^ ^53^ ^)^

This study represents by far the largest study of the relationship between sclerostin levels and CVD outcomes and related risk factors. We were able to replicate findings with respect to CVD risk factors across two separate cohorts, providing strong evidence of association between sclerostin and CVD risk factors for which only weak evidence existed previously, namely DM and HDL cholesterol. In addition, use of the LURIC cohort, which was recruited on the basis of high CAD risk and included outcomes from coronary angiograms, enabled well‐powered analysis of the relationship between sclerostin and detailed measures of CAD. As well as resulting in new insights into the relationship between sclerostin and CAD, availability of data for CVD risk factors in the same cohort enabled us to examine their contribution to the influence of sclerostin on CAD risk.

In terms of limitations, as stated above, given this was a cross‐sectional study, it is difficult to infer causality. That said, outcomes such as cardiac death were recorded prospectively after collection of baseline venesection samples used for sclerostin, providing some support for a causal relationship between sclerostin levels and CAD risk found in LURIC. LURIC represents a selected population, since participants were recruited on the basis of relatively high risk of CAD and so there may be some limitations in applying findings based on this cohort alone, such as relationships with CAD risk, to the wider population. ALSPAC mothers are more representative of the general population of women of reproductive age in the South West of England in the early 1990s. However, participating mothers with cIMT measures have some differences compared with the overall cohort recruited initially, such as being older, less likely to smoke, and a lower pre‐pregnancy BMI.^(^ ^32^ ^)^

A complete case analysis approach was used, which can lead to bias if data are not missing at random. However, sclerostin measures and outcome data were collected contemporaneously in both cohorts, minimizing the risk of bias due to loss to follow‐up. In LURIC, sclerostin and outcomes were collected at baseline at the point of recruitment, and although sclerostin measures were unavailable in approximately 30%, this was due to insufficient sample volume, which we assume occurred at random (the DiaSorin method for measuring sclerostin requires a relatively high sample volume of at least 220 μL). ALSPAC data was collected many years after inception of the cohort. However, although those attending the ALSPAC research clinic on which the present study was based had differences in age, smoking, and BMI compared with the original cohort, this is unlikely to have introduced bias given results were unchanged after we adjusted for these factors .

In terms of further limitations, the lack of detailed information about use of medications prevented us from examining their contribution to observed associations between sclerostin and risk factors such as diabetes mellitus. Furthermore, no censoring was performed in LURIC for all‐cause mortality; however, this is unlikely to have affected findings for CVD‐related mortality, given the lack of any known relationship between sclerostin levels and mortality from other causes. Finally, LURIC employed a novel automated DiaSorin chemiluminescent assay to measure sclerostin, which produced considerably lower values compared with the long‐established Biomedica ELISA used in ALSPAC, presumably due to the lack of detection of sclerostin fragments.^(^ ^29^ ^)^ However, despite these differences, meta‐analyses revealed similar associations between sclerostin and CVD risk factors in the two cohorts, with only associations between sclerostin and eGFR showing evidence of heterogeneity.

In conclusion, having examined associations between sclerostin and CVD and associated risk factors, we found that sclerostin is associated with increased CAD severity and mortality in LURIC. The former was partly explained by a relationship between higher sclerostin levels and CVD risk factors, namely DM, reduced eGFR, higher TGs, and lower HDL cholesterol, which were found to be related to sclerostin in meta‐analyses combining summary data from LURIC and ALSPAC. These findings are consistent with the suggestion that inhibition of osteoblast function as a result of higher sclerostin levels contributes to bone loss found in chronic kidney disease–mineral and bone disorder (CKD‐MBD),^(^ ^34^ ^)^ and point to a similar role in the development of osteoporosis associated with DM. Although our results are difficult to reconcile with trial evidence that sclerostin inhibition with romosozumab increases, rather than reduces, the risk of CVD events,^(^ ^5^ ^)^ care should be taken in extrapolating our findings given the mean age of the two cohorts is somewhat younger than the study populations of previous romosozumab trials. Given the importance of understanding the relationship between sclerostin and CVD risk for evaluating the safety of emerging osteoporosis therapy, our results highlight the need to explore these relationships in more detail, such as the use of Mendelian randomization, where further studies are required given the conflicting findings from investigations performed to date.^(^ ^52^, ^53^ ^)^

## Disclosures

MF receives salary funding from Wellcome Trust (project grant ref 209233). GDS, JZ, DAL, and JHT work in or are affiliated with a unit that receives funding from the UK Medical Research Council (MC_UU_00011/1, MC_UU_00011/4, MC_UU_00011/16). DAL is a National Institute of Health Research Senior Investigator (NF‐0616‐10102) and BHF Chair of Cardiovascular Science and Clinical Epidemiology (CH/F/20/90003). IG reports employment with Boehringer Ingelheim International GmbH, outside the submitted work. WM reports employment with Synlab Holding Deutschland GmbH, received grants from Abbott Diagnostics, grants and personal fees from Aegerion Pharmaceuticals, grants and personal fees from AMGEN, grants and personal fees from AstraZeneca, grants and personal fees from BASF, grants and personal fees from Danone Research, personal fees from MSD, grants and personal fees from Sanofi, grants and personal fees from Siemens Diagnostics, and personal fees from Synageva, all outside the submitted work.

## Author Contributions

**George Davey Smith:** conceptualization, funding Acquisition, writing original draft, writing review editing.

### Peer Review

The peer review history for this article is available at https://publons.com/publon/10.1002/jbmr.4467.

## Supporting information


**Appendix** **S1.** Supporting Information

## Data Availability

Data sharing is not applicable to this article as no new data were created or analyzed in this study.
