# Supplementary material for: Circulating Sclerostin Levels Are Positively Related to Coronary Artery Disease Severity and Related Risk Factors
Source: J Bone Miner Res. 2021 Dec 9;37(2):273–84. doi: 10.1002/jbmr.4467 (PMC9377011; doi:10.1002/jbmr.4467)
Supplement: Supplementary file 1 — Appendix S1. Supporting Information [file JBMR-37-273-s001.pdf]

Circulating sclerostin levels are positively related to coronary artery disease severity and related risk factors

M Frysz[1,5], I Gergei[2,3], H Scharnagl[4], G Davey Smith[5], J Zheng[5], DA Lawlor[5,6], M Herrmann[4], W Maerz[2,4,7], JH Tobias[1,5]

Supplementary Tables and Figures

Supplementary Table 1: Sclerostin versus clinical risk factors - additional adjustment for fasting glucose.

			LURIC N=2054		ALSPAC N=2977	
**Exposure**	**Outcome**	**Model**	**β (95% CI)**	**p**	**β (95% CI)**	**p**
Sclerostin	eGFR	2	-0.29 (-0.33,-0.25)	<0.001	-0.11 (-0.15,-0.08)	<0.001
Sclerostin	eGFR	3	-0.29 (-0.33,-0.25)	<0.001	-0.11 (-0.15,-0.08)	<0.001
			**OR (95% CI)**		**OR (95% CI)**	
Sclerostin	Hypertension	2	1.04 (0.93,1.17)	0.459	1.20 (1.03,1.41)	0.021
Sclerostin	Hypertension	3	1.03 (0.92,1.15)	0.592	1.20 (1.02,1.40)	0.025

Table shows results of linear/logistic regression analysis. Results are SD change in outcome/ odds of outcome per SD increase in sclerostin, 95% CI and p value. CI: Confidence Interval, eGFR: estimated Glomerular filtration rate. Model 2: adjusted for age and ethnic group (ALSPAC) and sex (LURIC), BMI, smoking, social deprivation. Model 3: Model 2 + fasting glucose (continuous measure).

Supplementary Table 2: Univariate associations between sclerostin and other risk factors in ALSPAC and LURIC

		LURIC N=2054			ALSPAC N=3015	
**Exposure**	**Outcome**	**β (95% CI)**	**p**		**β (95% CI)**	**p**
**sex**	Sclerostin	-0.38 (-0.47,-0.29)	<0.001			
**Age (years)**	Sclerostin	0.20 (0.16,0.24)	<0.001	**Age (years)**	0.19 (0.16,0.23)	<0.001
**BMI (kg/m2)**	Sclerostin	0.01 (-0.03,0.06)	0.504	**BMI (kg/m2)**	0.01 (-0.02,0.05)	0.536
**ex-smoker***	Sclerostin	0.25 (0.16,0.35)	<0.001	**Smoking (no vs yes)**	0.01 (-0.06,0.08)	0.791
**active smoker***	Sclerostin	-0.16 (-0.15,-0.01)	<0.001			
**Regional purchasing index**	Sclerostin	-0.03 (-0.07,0.04)	0.208	**Townsend score 2**	0.04 (-0.05,0.15)	0.360
				**Townsend score 3**	-0.06 (-1.16,0.04)	0.254
				**Townsend score 4**	0.06 (-0.04,0.16)	0.271
				**Townsend score 5**	-0.02 (-0.17,0.13)	0.818

Table shows results of linear regression analysis. Results are SD change in outcome per SD change in exposure/difference in exposure level in case of categorical exposures, 95% CI and p value.

*results attenuated following adjustment for age and sex;

ex-smoker [adjusted beta 0.11, CI(0.01, 0.21) p = 0.034]

active smoker [adjusted beta -0.10, CI(-0.22, 0.02) p = 0.114]

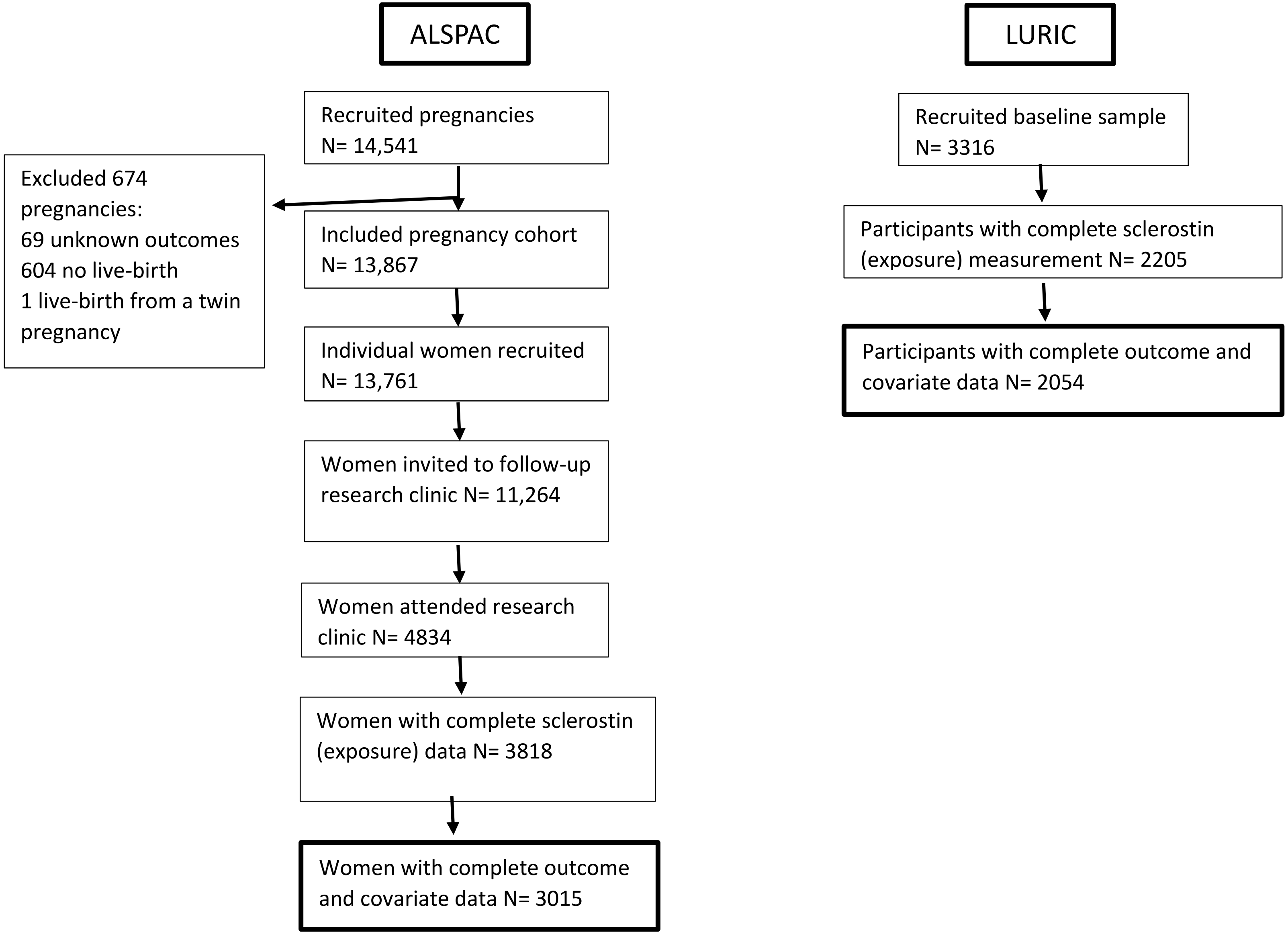

Supplementary Figure 1: Flow diagram showing the distribution of ALSPAC and LURIC participants from the recruitment to the present study population.

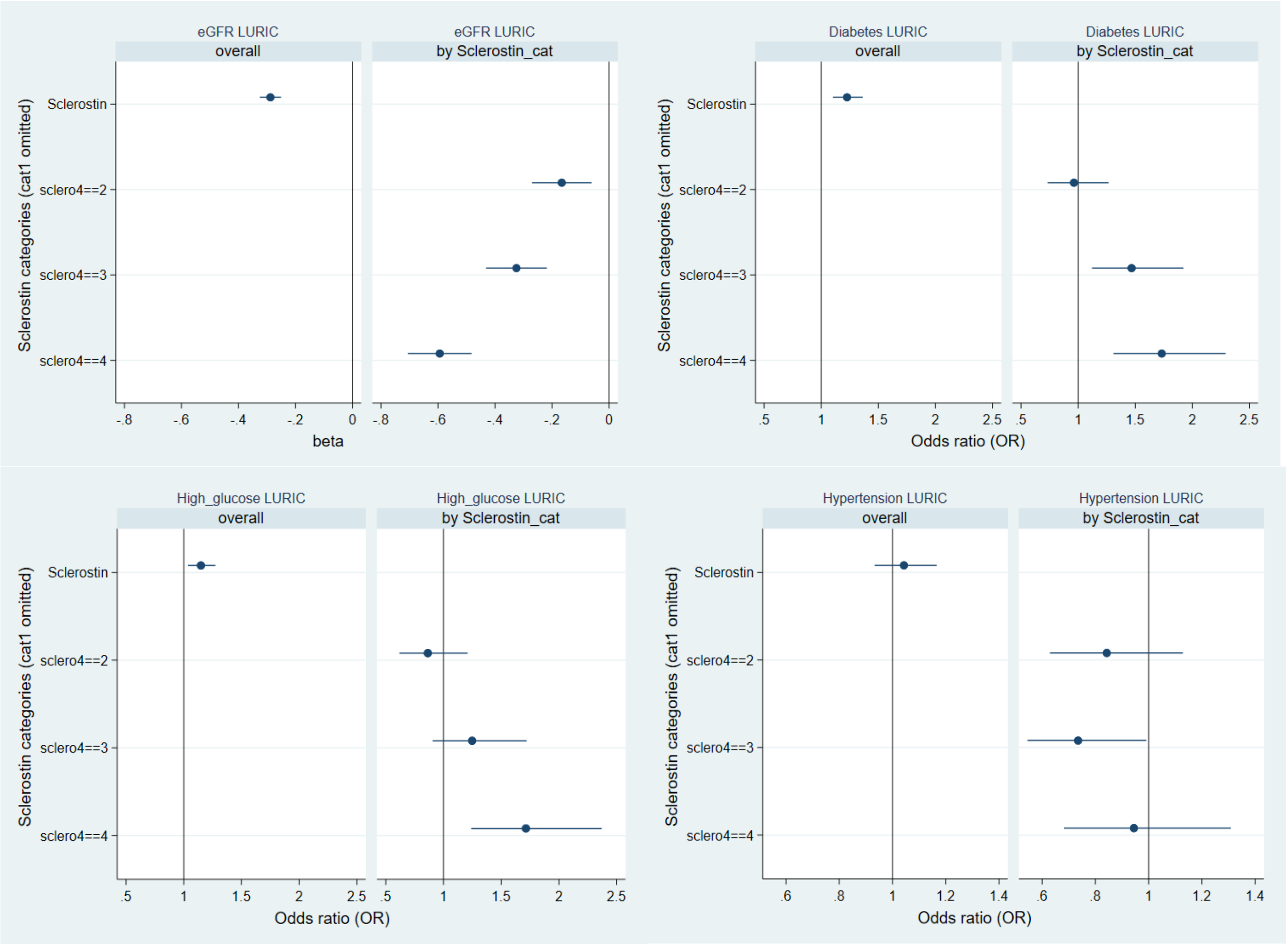

Supplementary Figure 2: Associations between sclerostin versus clinical risk factors (LURIC). Overall sclerostin (continuous measure) vs categorical sclerostin.

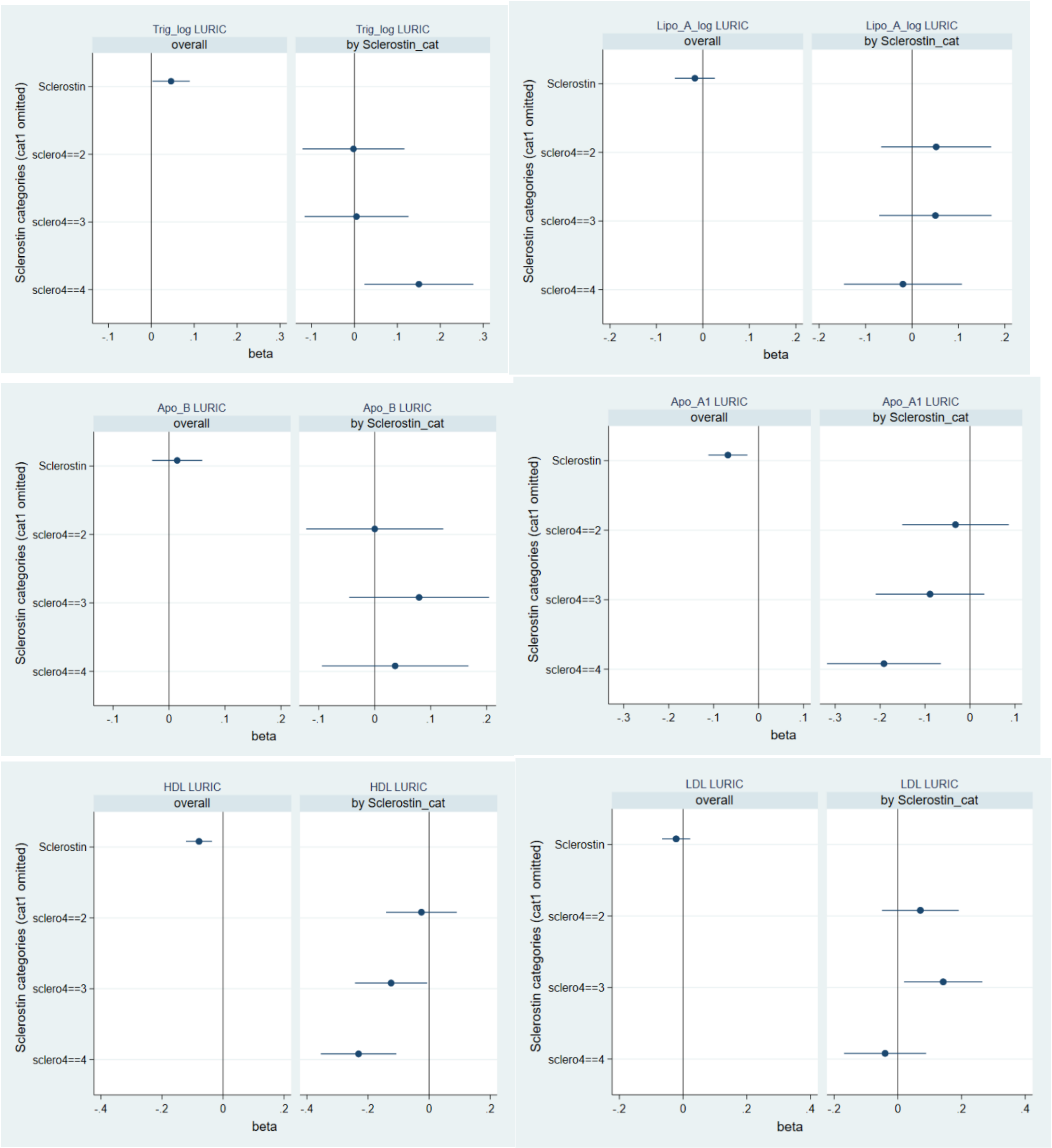

Supplementary Figure 3: Associations between sclerostin versus lipids (LURIC). Overall sclerostin (continuous measure) vs categorical sclerostin.

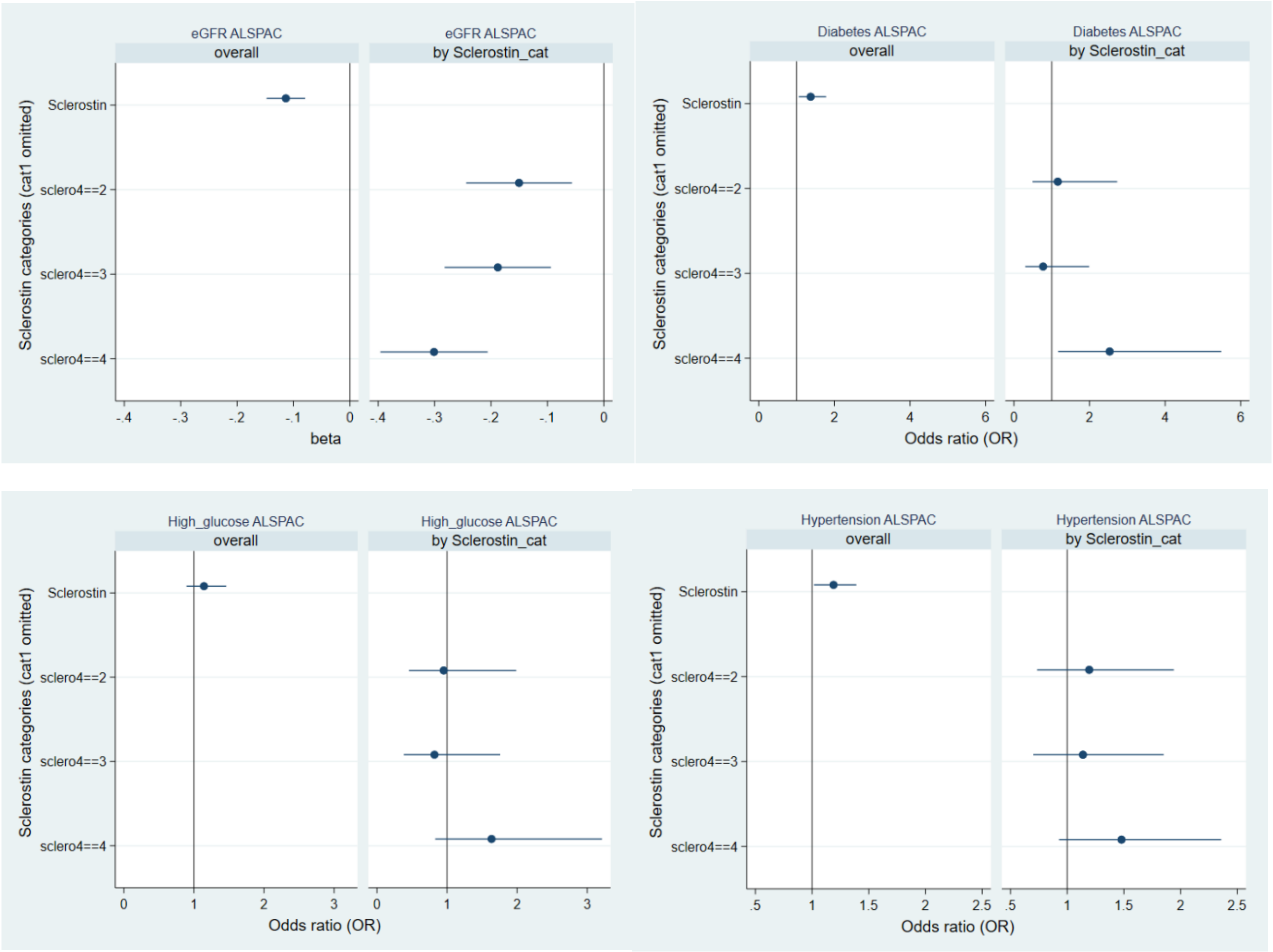

Supplementary Figure 4: Associations between sclerostin versus clinical risk factors (ALSPAC). Overall sclerostin (continuous measure) vs categorical sclerostin.

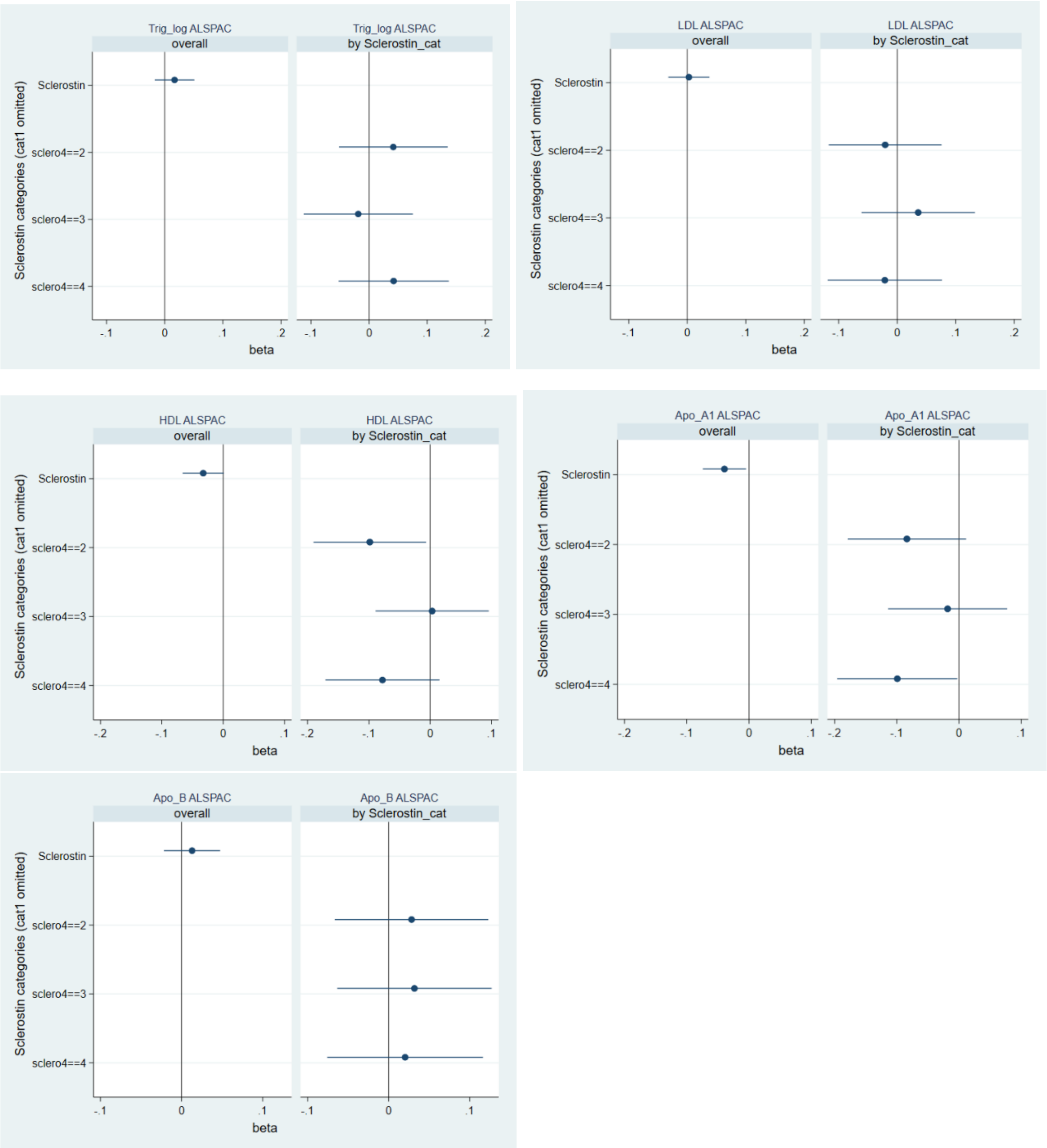

Supplementary Figure 5: Associations between sclerostin versus lipids (ALSPAC). Overall sclerostin (continuous measure) vs categorical sclerostin.

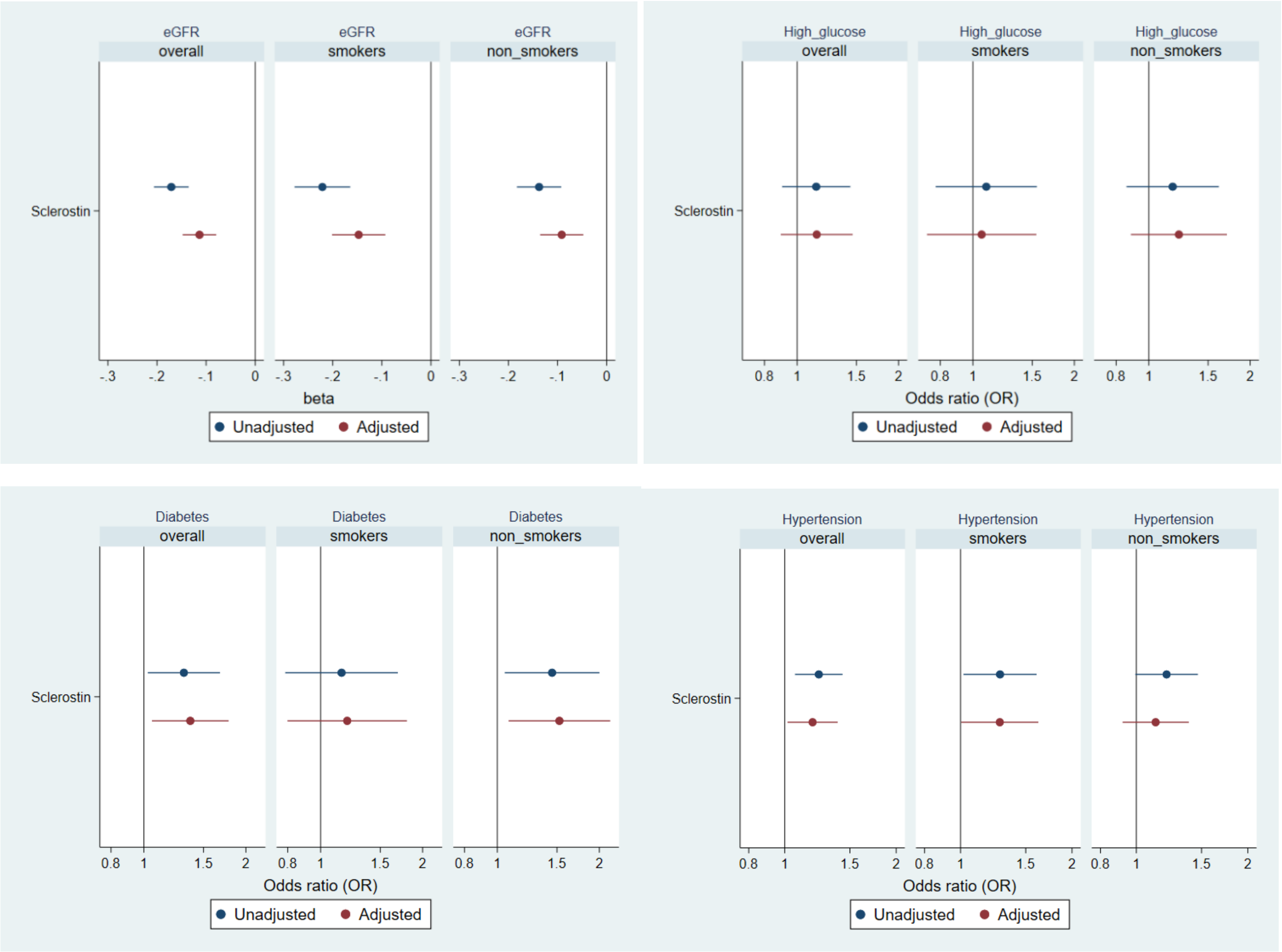

Supplementary Figure 6: Associations between sclerostin and clinical risk factors by smoking status in ALSPAC mothers.

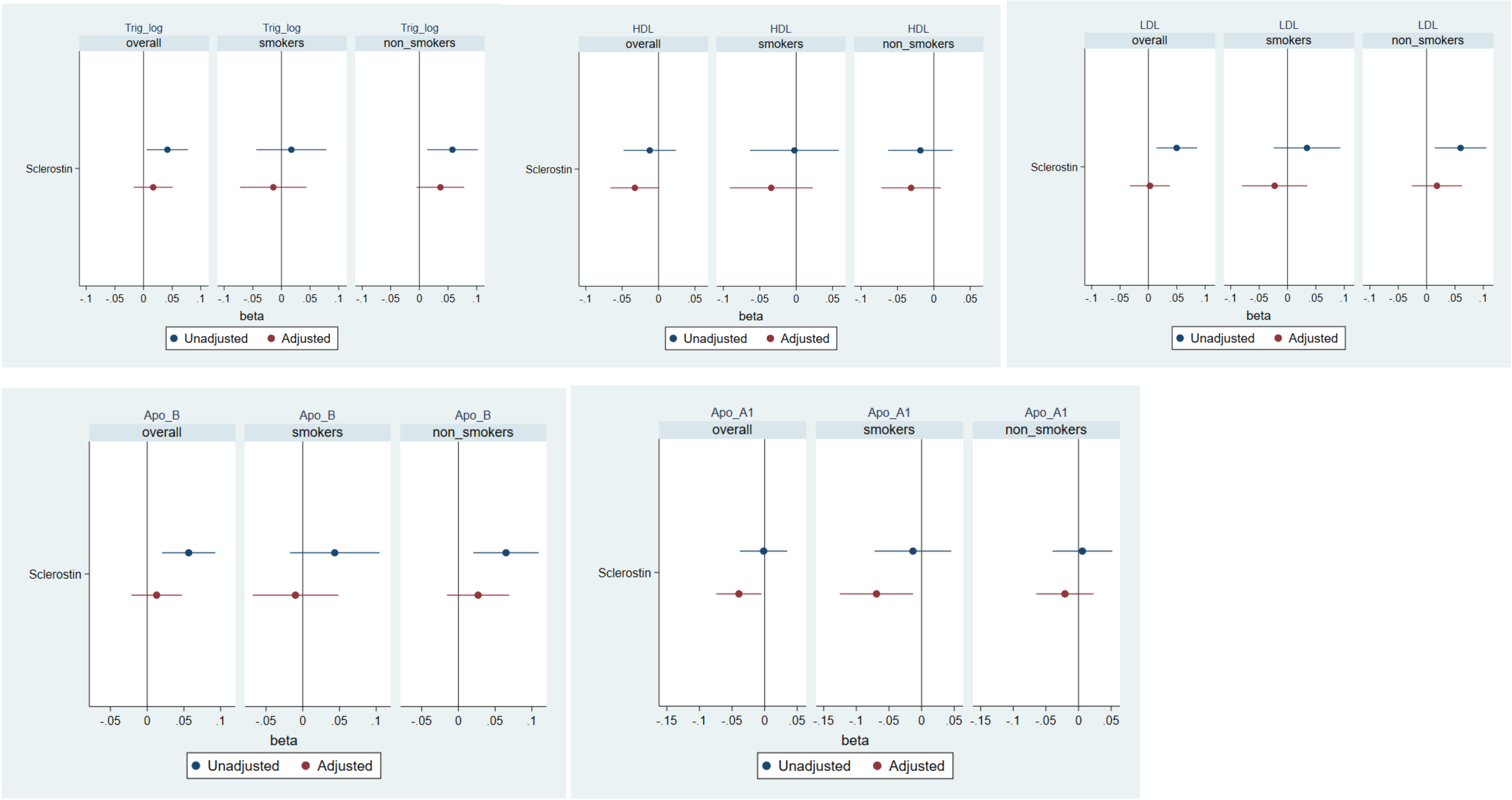

Supplementary Figure 7: Associations between sclerostin versus lipids stratified by smoking status in ALSPAC.

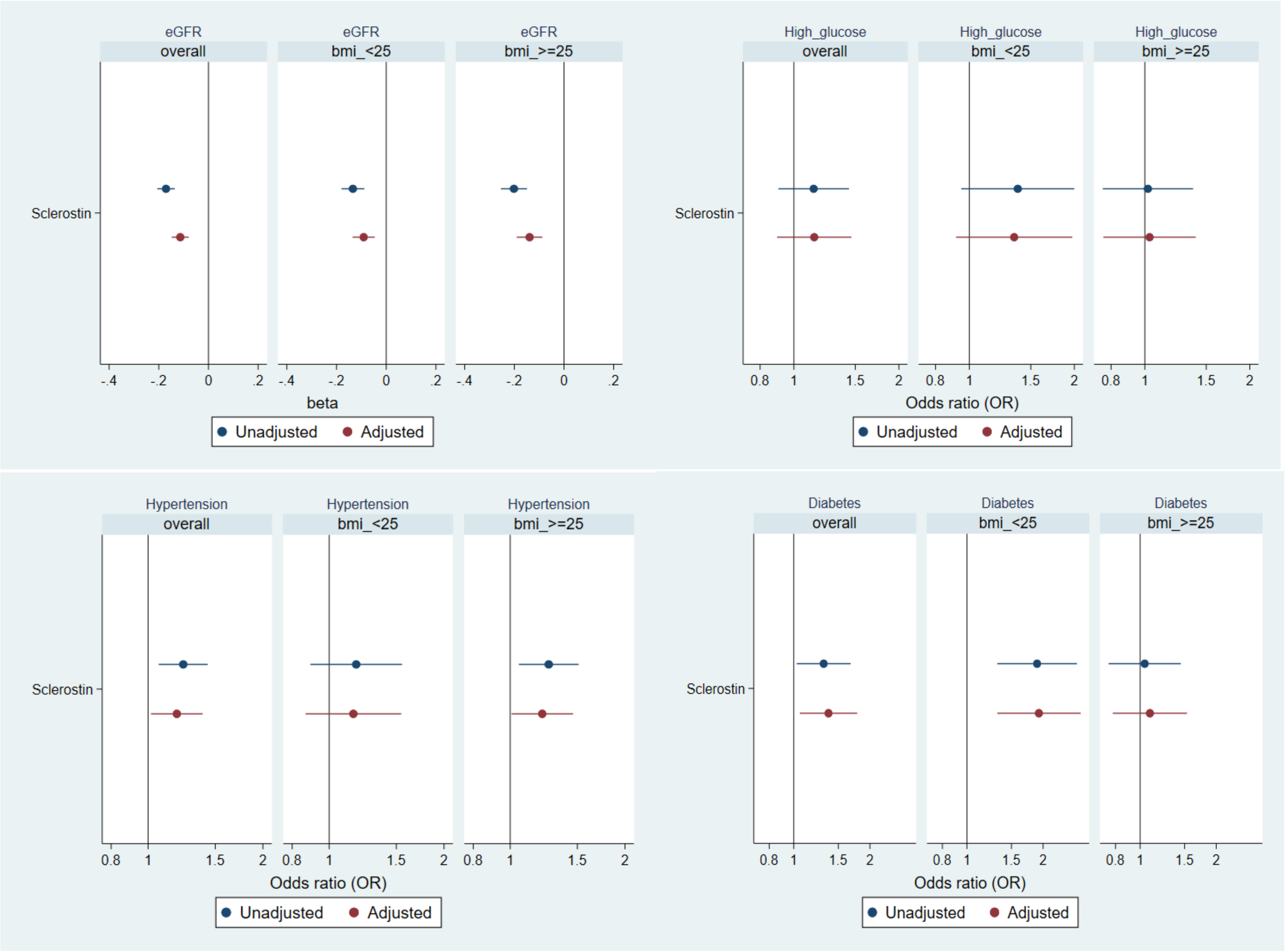

Supplementary Figure 8: Associations between sclerostin and clinical risk factors stratified by BMI category in ALSPAC.

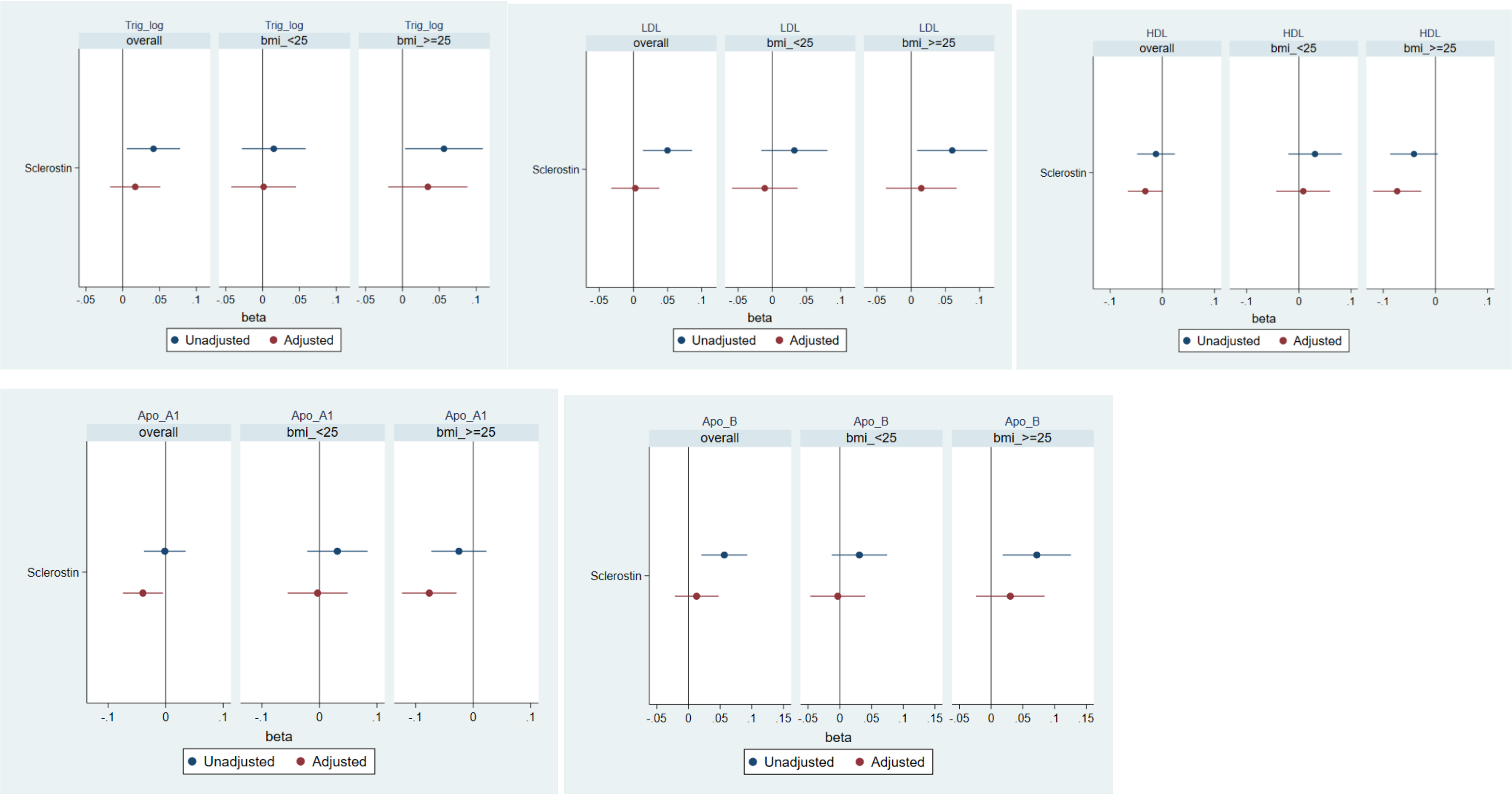

Supplementary Figure 9: Associations between sclerostin versus lipids stratified by BMI category in ALSPAC.

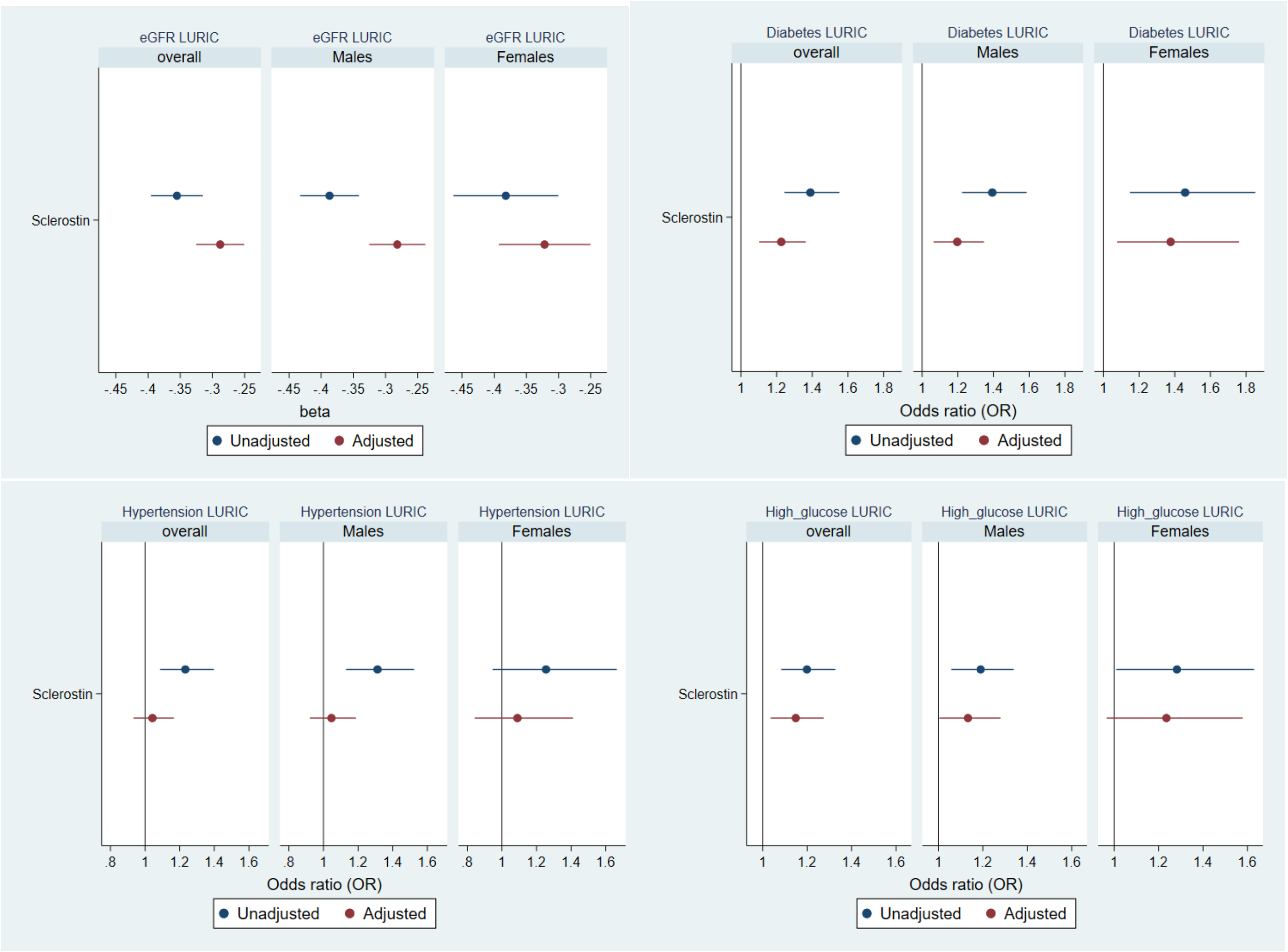

Supplementary Figure 10: Associations between sclerostin and clinical risk factors stratified by sex in LURIC.

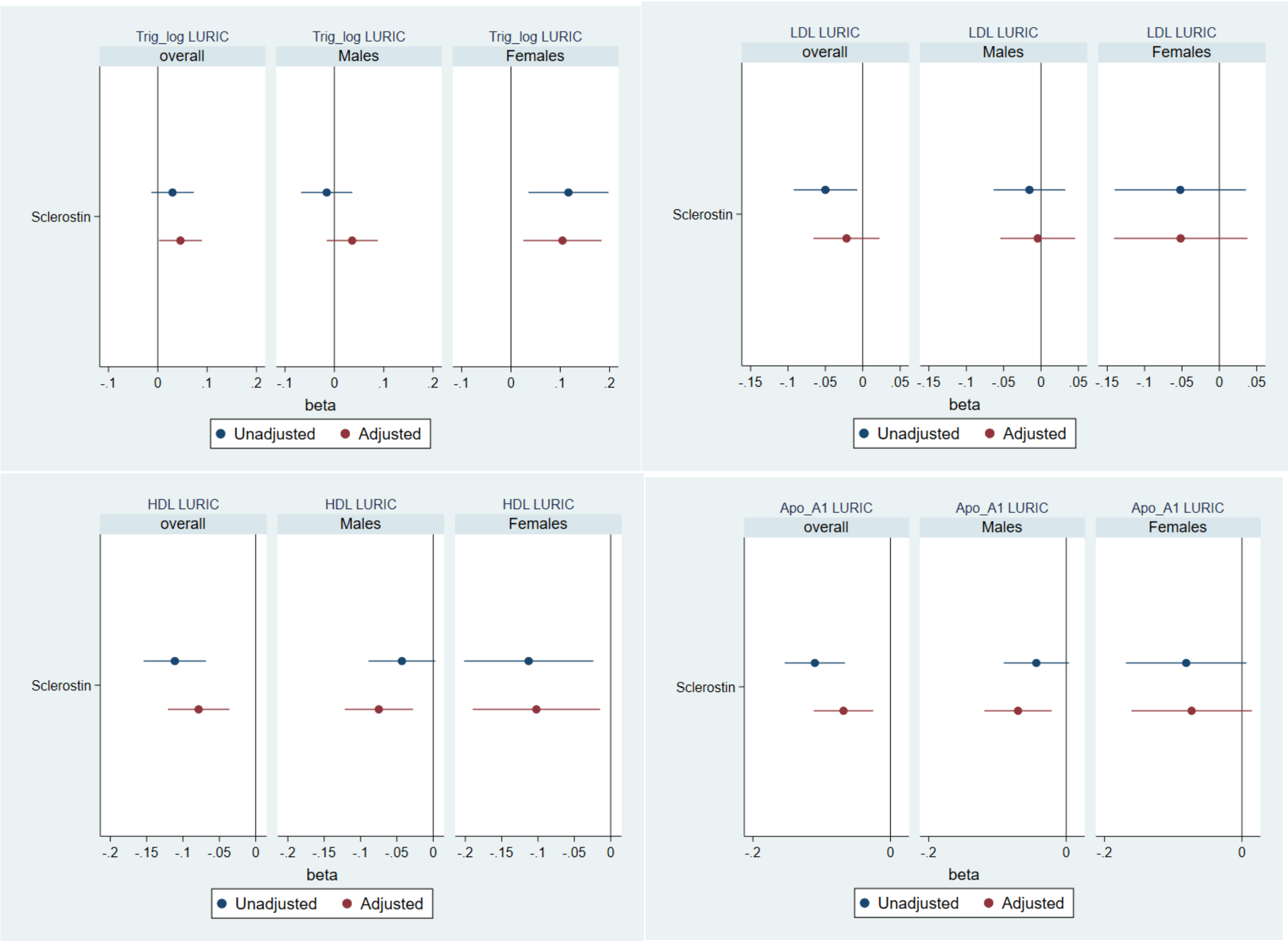
Trig_log LURIC
overall
Trig_log LURIC
Males
Trig_log LURIC
Females
Sclerostin
-.1 0 .1 .2
beta
Unadjusted
Adjusted
LDL LURIC
overall
LDL LURIC
Males
LDL LURIC
Females
Sclerostin
-.15 -.1 -.05 0 .05
beta
Unadjusted
Adjusted
HDL LURIC
overall
HDL LURIC
Males
HDL LURIC
Females
Sclerostin
-.2 -.15 -.1 -.05 0
beta
Unadjusted
Adjusted
Apo_A1 LURIC
overall
Apo_A1 LURIC
Males
Apo_A1 LURIC
Females
Sclerostin
-.2 0
beta
Unadjusted
Adjusted

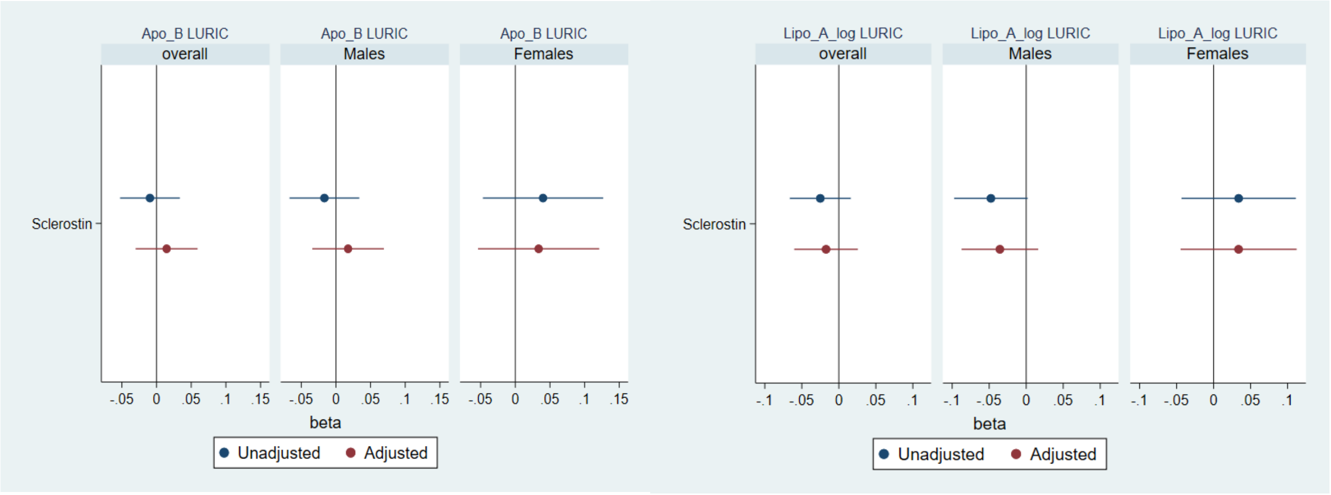

Supplementary Figure 11: Associations between sclerostin versus lipids stratified by sex in LURIC.
